# Supplementary material for: Addressing food insecurity in early childhood programs through a health equity lens: A qualitative case study of Brazil’s Criança Feliz program
Source: PLoS One. 2025 Jul 28;20(7):e0329310. doi: 10.1371/journal.pone.0329310 (PMC12303329; doi:10.1371/journal.pone.0329310)
Supplement: S1 Table — (DOCX) [file pone.0329310.s001.docx]

**Supplementary Table S1.**

Criança Feliz Program national guidelines following the Template for Intervention Description (TIDieR)

| **Intervention** | The *Criança Feliz Program* (PCF) aims to develop responsive parenting skills to provide early learning opportunities as a way to improve care for their children. |
| --- | --- |
| **Why** | Integrated early childhood education programs play a crucial role in promoting equity at an earlier stage in life. The PCF is a multisectoral nurturing care program that specifically targets the most vulnerable communities. Its ultimate goal is to assist in reducing poverty, inequities, and violence within the country. |
| **When** | The design of the PCF is based on two principles:  Home visits aiming to provide early stimulation and enhance early childhood outcomes. These visits are based on evidence from the Care for Child Development (CCD) curriculum, which focuses on early stimulation and promoting parenting skills. Pregnant individuals receive one visit per month, while caregivers of children under 36 months in socially vulnerable or at-risk situations receive weekly visits. Children under 72 months with disabilities receive biweekly or monthly visits. Additionally, children under 72 months who have lost at least one caregiver during the COVID-19 pandemic receive weekly visits from ages 0-36 months and biweekly visits from ages 36-72 months.  Complementary multisectoral actions aiming to address social determinants of health of families by strengthening cross sectoral collaborations among social assistance, health, education, culture, human rights, and children's rights. These efforts are focused on promoting a nurturing care environment. At each level of government, the PCF is expected to establish a Multisectoral Management Committee (MMC) responsible for coordinating the multisectoral actions. |
| **Who** | The PCF is implemented through a multi-level strategy involving the three levels of government (federal, state, and municipal) through the Unified Social Assistance System (SUAS).  At the federal level, the Ministry of Development and Social Assistance, Family, and Fight against Hunger is responsible for coordinating the national PCF implementation, providing support to states, and developing training strategies for facilitators, supervisors, and home visitors.  At the state level, the technical team is formed by a coordinator and facilitators who are responsible for supporting the municipal teams. The state PCF teams are tasked with providing technical assistance to municipalities, including awareness-raising initiatives, monitoring, mobilization, and training of municipal supervisors. At the municipal level, municipal technical teams coordinate PCF implementation. The PCF municipal team is formed by at least one municipal coordinator (optional), supervisors, and home visitors. Higher education is required for the municipal coordinator and the supervisors, while the home visitors must have at least completed high school. The supervisors oversee the home visitors, which is essential for maintaining PCF home visiting implementation fidelity and the operationalization of the necessary complementary multisectoral actions. The home visits must be planned and carried out by the home visitors, and monitored by the supervisors. |
| **How** | Organizational: In municipalities, the PCF teams should be housed in the Social Assistance Reference Centers (CRAS/SUAS). CRAS are responsible for providing various social assistance services and benefits to vulnerable families in a territory.  Caseload: The PCF municipal team is formed by at least one municipal coordinator (optional), supervisors (one for up to 15 home visitors), and home visitors (one for every 34 families).  Initial training: The initial training package consists of 80 hours, with 40 hours dedicated to the Care for Child Development (CCD) curriculum and 40hs in the PCF Home Visiting Guide. Training is conducted through a multi-level training cascade, where facilitators at the federal and state levels are the master trainers and are responsible to support municipalities in the initial training of home visitors and supervisors. |
| **When and Where** | Program inception: PCF was launched in 2016. By 2022, it had been implemented in 3,028 municipalities and surpassed the milestone of 57 million visits to vulnerable families.  Monthly target goals: Each municipality has a predefined monthly home visits target. |
| **How much** | National funding covers training and technical assistance costs at national and state levels. Municipalities receive a monthly reimbursement based on the achievement of their monthly home visits targets. The reimbursement cost per person visited is R$75 (about US$13.8*). The PCF had a budget of about US$77.7 million in 2022. |
| * Dollar average value in 2022 equals 5,40 reals. Abbreviations: CHW – Community Health Workers, CCD - Child Care and Development, CRAS - Social Assistance Reference Center, MMC –Multisectoral Management Committee, PCF - *Criança Feliz Program*, Unified Social Assistance System (SUAS). | |
