# Supplementary material for: Addressing food insecurity in early childhood programs through a health equity lens: A qualitative case study of Brazil’s Criança Feliz program
Source: PLoS One. 2025 Jul 28;20(7):e0329310. doi: 10.1371/journal.pone.0329310 (PMC12303329; doi:10.1371/journal.pone.0329310)
Supplement: S3 Table — (DOCX) [file pone.0329310.s003.docx]

**Supplementary Table S3**

Socio-demographic characteristics of the professionals interviewed in the *Criança Feliz Program* (PCF), 2021-2022.

| Characteristics | **Total** | | **Campo Grande** | | **Cuité** | | **Brasilia** | | **Fortaleza** | | **São Paulo** | |
| --- | --- | --- | --- | --- | --- | --- | --- | --- | --- | --- | --- | --- |
|  | **n** | **%** | **n** | **%** | **n** | **%** | **n** | **%** | **n** | **%** | **n** | **%** |
| **Participants** |  |  |  |  |  |  |  |  |  |  |  |  |
| PCF Municipal coordinators | 38 | 25.9 | 1 | 6.3 | 2 | 20.0 | 5 | 17.2 | 7 | 17.5 | 23 | 44.2 |
| PCF Supervisors | 30 | 20.4 | 3 | 18.8 |  | 0.0 | 5 | 17.2 | 8 | 20.0 | 14 | 26.9 |
| PCF Home Visitors | 69 | 46.9 | 8 | 50.0 | 5 | 50.0 | 16 | 55.2 | 25 | 62.5 | 15 | 28.8 |
| Municipal managers from sectors working with PCF | 10 | 6.8 | 4 | 25.0 | 3 | 30.0 | 3 | 10.3 | - | - | - | - |
| **Time in the position** |  |  |  |  |  |  |  |  |  |  |  |  |
| No information | 3 | 2.0 | - | - | - | - | 2 | 6.9 | 1 | 2.5 | - | - |
| 0 to 2 years | 87 | 59.2 | 6 | 37.5 | 4 | 40.0 | 27 | 93.1 | 28 | 70.0 | 22 | 42.3 |
| > 2 to 3 years | 25 | 17.0 | 5 | 31.3 | 4 | 40.0 | - | - | 3 | 7.5 | 13 | 25.0 |
| >= 4 years | 32 | 21.8 | 5 | 31.3 | 2 | 20.0 | - | - | 8 | 20.0 | 17 | 32.7 |
| **Time working with public policies** |  |  |  |  |  |  |  |  |  |  |  |  |
| No information | 4 | 2.7 | - | - | - | - | - | - | 4 | 10.0 | - | - |
| 1 to 4 years | 56 | 38.1 | 4 | 25.0 | 6 | 60.0 | 26 | 89.7 | 7 | 17.5 | 13 | 25.0 |
| 5 to 11 years | 41 | 27.9 | 4 | 25.0 | 3 | 30.0 | 1 | 3.4 | 10 | 25.0 | 23 | 44.2 |
| >= 12 years | 46 | 31.3 | 8 | 50.0 | 1 | 10.0 | 2 | 6.9 | 19 | 47.5 | 16 | 30.8 |
| **Skin Color** |  |  |  |  |  |  |  |  |  |  |  |  |
| No information | 6 | 4.1 | - | - | - | - | - | - | 6 | 15.0 | - | - |
| Yellow | 6 | 4.1 | 1 | 6.3 | 1 | 10.0 | - | - | - | - | 4 | 7.7 |
| White | 44 | 29.9 | 4 | 25.0 | 3 | 30.0 | 9 | 31.0 | 9 | 22.5 | 19 | 36.5 |
| Brown/Black | 91 | 61.9 | 11 | 68.8 | 6 | 60.0 | 20 | 69.0 | 25 | 62.5 | 29 | 55.8 |
| **Gender** |  |  |  |  |  |  |  |  |  |  |  |  |
| Female | 131 | 89.1 | 14 | 87.5 | 9 | 90.0 | 24 | 82.8 | 36 | 90.0 | 48 | 92.3 |
| Male | 16 | 10.9 | 2 | 12.5 | 1 | 10.0 | 5 | 17.2 | 4 | 10.0 | 4 | 7.7 |
| **Age groups, in years** |  |  |  |  |  |  |  |  |  |  |  |  |
| No information | 6 | 4.1 | - | - | - | - | - | - | 6 | 15.0 | - | - |
| up to 25 | 8 | 5.4 | - | - | - | - | 7 | 24.1 | 1 | 2.5 | - | - |
| 26 to 37 | 56 | 38.1 | 5 | 31.3 | 4 | 40.0 | 13 | 44.8 | 12 | 30.0 | 22 | 42.3 |
| 38 to 41 | 18 | 12.2 | 3 | 18.8 | 2 | 20.0 | 4 | 13.8 | 3 | 7.5 | 6 | 11.5 |
| 42 or more | 59 | 40.1 | 8 | 50.0 | 4 | 40.0 | 5 | 17.2 | 18 | 45.0 | 24 | 46.2 |
| Education |  |  |  |  |  |  |  |  |  |  |  |  |
| No information | 2 | 1.4 | - | - | - | - | - | - | 2 | 5.0 | - | - |
| High school | 20 | 13.6 | - | - | 4 | 40.0 | 2 | 6.9 | 14 | 35.0 | - | - |
| College | 72 | 49.0 | 7 | 43.8 | 4 | 40.0 | 16 | 55.1 | 14 | 35.0 | 31 | 59.6 |
| Postgraduation | 53 | 36.1 | 9 | 56.3 | 2 | 20.0 | 11 | 37.9 | 10 | 25.0 | 21 | 40.4 |
| **College Degree Area*** |  |  |  |  |  |  |  |  |  |  |  |  |
| Health | 40 | 37.0 | 11 | 44.0 | 2 | 20.0 | 4 | 14.3 | 7 | 30.4 | 16 | 59.3 |
| Sciences | 3 | 2.8 | - | - | 1 | 10.0 | 2 | 7.1 | - | - | - | - |
| Social Sciences | 24 | 22.2 | 6 | 24.0 | 1 | 10.0 | 7 | 25.0 | 9 | 39.1 | 1 | 3.7 |
| Education | 22 | 20.4 | 6 | 24.0 | - | - | 6 | 21.4 | 2 | 8.7 | 8 | 29.6 |
| Administration | 2 | 1.9 | - | - | - | - | - | - | 1 | 4.3 | 1 | 3.7 |
| Others | 17 | 15.7 | 2 | 8.0 | 1 | 10.0 | 9 | 32.1 | 4 | 17.4 | 1 | 3.7 |

*one informant can have multiple college degrees. Health (Nursing/ Nutrition/Physical Education/Psychology), Sciences (Biology, Chemistry), Social Sciences (Social Sciences, Social Work, Philosophy), Education, Administration (Business and Public Administration), Others (Human resources, Law, International Affairs, Digital Marketing, Secretary, Languages, Visual Arts, Accounting).
