# Supplementary material for: Addressing food insecurity in early childhood programs through a health equity lens: A qualitative case study of Brazil’s Criança Feliz program
Source: PLoS One. 2025 Jul 28;20(7):e0329310. doi: 10.1371/journal.pone.0329310 (PMC12303329; doi:10.1371/journal.pone.0329310)
Supplement: S4 Table — (DOCX) [file pone.0329310.s004.docx]

**Supplementary Table S4**

Socio-demographic characteristics of the families interviewed in the *Criança Feliz Program* (PCF), 2021-2022.

| **Categories** | **Total** | | **Campo Grande** | | **Cuité** | | **Brasilia** | | **Fortaleza** | | **São Paulo*** | |
| --- | --- | --- | --- | --- | --- | --- | --- | --- | --- | --- | --- | --- |
|  | n | % | n | % | n | % | n | % | n | % | n | % |
| **PCF participant** |  |  |  |  |  |  |  |  |  |  |  |  |
| Children | 80 | 86.0 | 13 | 86.7 | 10 | 100.0 | 14 | 63.6 | 18 | 94.7 | 25 | 92.6 |
| Pregnant individuals | 9 | 9.7 | 2 | 13.3 | - | - | 4 | 18.2 | 1 | 5.3 | 2 | 7.4 |
| Both (child and pregnant) | 4 | 4.3 | - | - | - | - | 4 | 18.2 | - | - | - | - |
| **Informant** |  |  |  |  |  |  |  |  |  |  |  |  |
| Child’s mother | 81 | 86.2 | 15 | 100.0 | 10 | 100.0 | 13 | 59.1 | 18 | 94.7 | 25 | 89.3 |
| Pregnant individual | 5 | 5.3 | - | - | - | - | 4 | 18.2 | 1 | 5.3 | - | - |
| Child’s mother and pregnant | 6 | 6.4 | - | - | - | - | 4 | 18.2 | - | - | 2 | 7.1 |
| Father* | 2 | 2.1 | - | - | - | - | 1 | 4.5 | - | - | 1 | 3.6 |
| **Informant skin color** |  |  |  |  |  |  |  |  |  |  |  |  |
| No information | 3 | 3.2 | - | - | - | - | - | - | - | 15.8 | - | - |
| Yellow | 2 | 2.1 | 1 | 6.7 | - | - | - | - | 1 | 5.3 | - | - |
| White | 11 | 11.7 | - | - | 2 | 20.0 | 2 | 9.1 | - | - | 7 | 25.0 |
| Brown/Black | 78 | 83.0 | 14 | 93.3 | 8 | 80.0 | 20 | 90.9 | 15 | 78.9 | 21 | 75.0 |
| **Informant age, in years** |  |  |  |  |  |  |  |  |  |  |  |  |
| No information | 2 | 2.1 | - | - | - | - | - | - | 2 | 10.5 | - | 0.0 |
| 16 to 24 | 22 | 23.4 | 4 | 26.7 | 4 | 40.0 | 5 | 22.7 | 4 | 21.1 | 5 | 17.9 |
| 25 to 29 | 22 | 23.4 | 5 | 33.3 | 2 | 20.0 | 3 | 13.6 | 4 | 21.1 | 8 | 28.6 |
| 30 or more | 48 | 51.1 | 6 | 40.0 | 4 | 40.0 | 14 | 63.6 | 9 | 47.4 | 15 | 53.6 |
| **Informant Occupation** |  |  |  |  |  |  |  |  |  |  |  |  |
| No information | 1 | 1.1 | - | - | - | - | - | - | 1 | 5.3 |  | 0.0 |
| Working | 27 | 28.7 | 4 | 26.7 | 3 | 30.0 | 6 | 27.3 | 6 | 31.6 | 8 | 28.6 |
| Not working | 66 | 70.2 | 11 | 73.3 | 7 | 70.0 | 16 | 72.7 | 12 | 63.2 | 20 | 71.4 |
| **Informant Education** |  |  |  |  |  |  |  |  |  |  |  |  |
| Primary school | 31 | 33.0 | 7 | 46.7 | 3 | 30.0 | 4 | 18.2 | 6 | 31.6 | 11 | 39.3 |
| High school | 52 | 55.3 | 6 | 40.0 | 7 | 70.0 | 13 | 59.1 | 10 | 52.6 | 16 | 57.1 |
| College degree | 11 | 11.7 | 2 | 13.3 | - | - | 5 | 22.7 | 3 | 15.8 | 1 | 3.6 |
| **Number of people in the household** |  |  |  |  |  |  |  |  |  |  |  |  |
| 2 to 4 | 57 | 61.3 | 4 | 26.7 | 7 | 70.0 | 14 | 63.6 | 14 | 73.7 | 18 | 66.7 |
| 5 to 6 | 23 | 24.7 | 7 | 46.7 | 2 | 20.0 | 5 | 22.7 | 4 | 21.1 | 5 | 18.5 |
| 7 or more | 13 | 14.0 | 4 | 26.7 | 1 | 10.0 | 3 | 13.6 | 1 | 5.3 | 4 | 14.8 |
| **Receive Continuous Cash Benefit Program** |  |  |  |  |  |  |  |  |  |  |  |  |
| No | 78 | 83.9 | 12 | 80.0 | 9 | 90.0 | 15 | 68.2 | 18 | 94.7 | 24 | 88.9 |
| Yes | 15 | 16.1 | 3 | 20.0 | 1 | 10.0 | 7 | 31.8 | 1 | 5.3 | 3 | 11.1 |
| **Receive Bolsa Familia Program** |  |  |  |  |  |  |  |  |  |  |  |  |
| No | 28 | 30.1 | 2 | 13.3 | 1 | 10.0 | 9 | 40.9 | 8 | 42.1 | 8 | 29.6 |
| Yes | 65 | 69.9 | 13 | 86.7 | 9 | 90.0 | 13 | 59.1 | 11 | 57.9 | 19 | 70.4 |
| **Receive another government benefit** |  |  |  |  |  |  |  |  |  |  |  |  |
| No | 63 | 67.7 | 14 | 93.3 | 10 | 100.0 | 6 | 27.3 | 15 | 78.9 | 18 | 66.7 |
| Yes | 30 | 32.3 | 1 | 6.7 | - | - | 16 | 72.7 | 4 | 21.1 | 9 | 33.3 |
| **Food insecurity** |  |  |  |  |  |  |  |  |  |  |  |  |
| No | 41 | 44.1 | 4 | 26.7 | 6 | 60.0 | 9 | 40.9 | 13 | 68.4 | 9 | 33.3 |
| Yes | 52 | 55.9 | 11 | 73.3 | 4 | 40.0 | 13 | 59.1 | 6 | 31.6 | 18 | 66.7 |
| **Time Participating in the PCF Program** |  |  |  |  |  |  |  |  |  |  |  |  |
| No information | 5 | 5.4 | - | - | - | - | - | - | 5 | 26.3 |  |  |
| 6 to 13 months | 58 | 62.4 | 4 | 26.7 | 3 | 30.0 | 22 | 100.0 | 9 | 47.4 | 20 | 74.1 |
| 14 to 21 months | 14 | 15.1 | 5 | 33.3 | 2 | 20.0 | - | - | 4 | 21.1 | 3 | 11.1 |
| 22 months or more | 16 | 17.2 | 6 | 40.0 | 5 | 50.0 | - | - | 1 | 5.3 | 4 | 14.8 |

*Both parents participated in one of the interviews.
