# Supplementary material for: Addressing food insecurity in early childhood programs through a health equity lens: A qualitative case study of Brazil’s Criança Feliz program
Source: PLoS One. 2025 Jul 28;20(7):e0329310. doi: 10.1371/journal.pone.0329310 (PMC12303329; doi:10.1371/journal.pone.0329310)
Supplement: S5 Table — (DOCX) [file pone.0329310.s005.docx]

**Supplementary Table S5.**

Selected questions from the interview guide related to food insecurity, vulnerability, and equity according to the RE-AIM Framework dimensions (Reach, Effectiveness, Adoption, Implementation, Maintenance).

| **PCF teams and Managers (translated from Portuguese)**  **IMPLEMENTATION**   - Tell me how Criança Feliz works in your territory?   **Home visits & Intersectoral Actions**   - Tell us a little about the operation of home visits. Are there goals? Are there barriers? - Tell us a little about the operationalization of intersectoral actions in the context of the Happy Child Program? Can you give me some examples. ( *Barriers and Facilitators* ) - Is there systematized monitoring of intersectoral actions in the field?   **Multisectoral Actions (Bolsa Familia & Food Insecurity)**   - Are there programs aimed at the development of children in the municipality? How do they relate to the Happy Child Program? Is there some forwarding flow, protocol. - In relation to the Bolsa Familia Program, is there any interaction or integration of actions? If yes, tell or give examples. - Is there any action focused on food insecurity? How is this topic approached by you and your team? Examples.   **REACH & EQUITY**   - What is the socioeconomic profile of families receiving the PCF in your territory in terms of race and vulnerability? Percentage of blacks? How has the program dealt with families who live further away?   **Demands from families**   - What is the perception of families regarding the Happy Child Program? Are there any expectations of families that the program does not meet? |
| --- |
| **Caregivers (translated from Portuguese)**  **MULTISECTORAL ACTIONS**   - Have you ever been referred to any service through the Programa Criança Feliz? If yes, for which service? In what situation and why? - Is your child followed up at the UBS (Primary Healthcare Center)? How often? (For example, prenatal care, vaccination). At the UBS, do they discuss your child’s development? Has your child ever had any health problems? When and how did you find help? - Is your child in daycare? - Have you participated in other programs or public services? If yes, which ones? How did they work? - Besides the visits, has the Programa Criança Feliz helped your family in other ways? (For example: *Healthcare, Social assistance, Education)*   **FAMILIES’ NEEDS**   - Do you feel emotionally overwhelmed? If so, is this overload related to motherhood? - Do you have space in your home to play with your child? Do you pay rent? In your home, have you ever had problems with the water supply? Sewage? Electricity? - What were your expectations regarding the Programa Criança Feliz? Has the PCF offered everything you want? Are there any expectations that the program hasn’t met? Provide examples.   **COVID-19**   1. How has the pandemic changed your family? For example, if the child attended daycare, how did you deal with the closing of the schools? How was it to stay home with the child? (Use of punitive disciplines, for example). Has the child stopped going to the UBS, stopped taking vaccinations?   **FOOD INSECURITY**   - *If you receive a food basket: How often?* Is it sufficient to meet your family’s needs? - **“In the past 12 months the food that (I/we) bought just didn’t last, and (I/we) didn’t have money to get more” ( ) Yes ( ) No [1, 2]** - **“In the past 12 months, did (you/you or other adults in your household) ever cut the size of your meals or skip meals because there wasn't enough money for food?” ( ) Yes ( ) No [1, 2]** - If yes for both, do you believe that the Programa Criança Feliz or some other government program could help you with this situation? - If yes, how have you dealt with it? - Do you breastfeed? How long did you breastfeed? Why?   **SUSTAINABILITY**   - Would you change anything in the program? - How do you feel about continuing in the program? - Is there anything else you would like to share about your experience with the program? |
