# Supplementary material for: Addressing food insecurity in early childhood programs through a health equity lens: A qualitative case study of Brazil’s Criança Feliz program
Source: PLoS One. 2025 Jul 28;20(7):e0329310. doi: 10.1371/journal.pone.0329310 (PMC12303329; doi:10.1371/journal.pone.0329310)
Supplement: Attachment — Submitted filename: PLOS ONE - OCT24.pdf [file pone.0329310.s006.pdf]

PLOS ONE

Addressing Food Insecurity in Early Childhood Programs through a Health Equity Lens: A Qualitative Case Study of Brazil’s Criança Feliz Program.

--Manuscript Draft--

**Manuscript Number:**	PONE-D-24-37907
**Article Type:**	Research Article
**Full Title:**	Addressing Food Insecurity in Early Childhood Programs through a Health Equity Lens: A Qualitative Case Study of Brazil’s Criança Feliz Program.
**Short Title:**	Addressing Food Insecurity in Early Childhood Programs through a Health Equity Lens
**Corresponding Author:**	Gabriela Buccini, Ph.D.<br>University of Nevada Las Vegas<br>Las Vegas, NV UNITED STATES OF AMERICA
**Keywords:**	Food insecurity; early childhood; early childhood programs; equity
**Abstract:**	Background: Food insecurity (FI) is a public health crisis that negatively impacts early childhood health and development. Recommendations for establishing equitable actions to address FI in early childhood programming have not been developed globally. The Brazilian Criança Feliz Program (PCF), one of the largest early childhood home visiting programs worldwide, has faced implementation challenges while addressing FI. We aimed to (1) analyze programmatic barriers to addressing FI and (2) develop equity-focused recommendations to improve program implementation to mitigate FI.<br>Methods: Qualitative case study analyses of in-depth interviews with PCF implementation teams and families in five Brazilian municipalities. Participants shared their experience with PCF implementation quality, including questions related to FI. A three-stage rapid qualitative approach was used: (1) inductive thematic analysis identified central codes related to FI, (2) deductive approach to organize central codes within themes related to internal and external contexts of PCF operations, and (3) integration of findings into a set of equity-focused recommendations based on the four dimensions of the Getting to Equity (GTE) framework.<br>Results: 240 interviews were conducted. Internal programmatic barriers included lack of protocols for screening, referring, and following up with families struggling to access food as well as challenges to engage them in early learning activities. External programmatic barriers included family-level challenges (e.g. unrealistic support expected from PCF) and system-level challenges (e.g. bureaucracy in accessing safety nets). Ten equity-focused recommendations across GTE framework focused on improving program curriculum and protocols to mitigate FI and increasing individual and community capacity.<br>Conclusions: We documented barriers at the family, program, and system levels to address FI in the Brazilian PCF home visiting program. Barriers informed the generation of equity-focused programming recommendations to improve practices to address FI, not only for the PCF, but also for the global community implementing home visiting programs.
**Order of Authors:**	Gabriela Buccini, Ph.D.
	Poliana de Araújo Palmeira
	Ana Poblacion
	Muriel Bauermann Gubert
**Additional Information:**	
**Question**	**Response**
**Financial Disclosure**<br><br>Enter a financial disclosure statement that describes the sources of funding for the	Yes

work included in this submission. Review the submission guidelines for detailed requirements. View published research articles from *PLOS ONE* for specific examples.<br><br>This statement is required for submission and **will appear in the published article** if the submission is accepted. Please make sure it is accurate.<br><br>**Funded studies**<br>Enter a statement with the following details:<br>• Initials of the authors who received each award<br>• Grant numbers awarded to each author<br>• The full name of each funder<br>• URL of each funder website<br>• Did the sponsors or funders play any role in the study design, data collection and analysis, decision to publish, or preparation of the manuscript?<br><br>Did you receive funding for this work?	
Please add funding details.<br>as follow-up to "**Financial Disclosure**<br><br>Enter a financial disclosure statement that describes the sources of funding for the work included in this submission. Review the submission guidelines for detailed requirements. View published research articles from *PLOS ONE* for specific examples.<br><br>This statement is required for submission and **will appear in the published article** if the submission is accepted. Please make sure it is accurate.	The research was supported by "The Eunice Kennedy Shriver National Institute Of Child Health & Human Development of the National Institutes of Health" under Award Number R00HD097301 (PI: Buccini). The content is the sole responsibility of the authors and does not necessarily represent the official opinion of the National Institutes of Health. The funders had no role in study design, data collection and analysis, decision to publish, or preparation of the manuscript.

Powered by Editorial Manager® and ProduXion Manager® from Aries Systems Corporation

**Funded studies**<br>Enter a statement with the following details:<br>• Initials of the authors who received each award<br>• Grant numbers awarded to each author<br>• The full name of each funder<br>• URL of each funder website<br>• Did the sponsors or funders play any role in the study design, data collection and analysis, decision to publish, or preparation of the manuscript?<br><br>Did you receive funding for this work?"	
Please select the country of your main research funder (please select carefully as in some cases this is used in fee calculation).<br>as follow-up to "**Financial Disclosure**<br><br>Enter a financial disclosure statement that describes the sources of funding for the work included in this submission. Review the submission guidelines for detailed requirements. View published research articles from *PLOS ONE* for specific examples.<br><br>This statement is required for submission and **will appear in the published article** if the submission is accepted. Please make sure it is accurate.<br><br>**Funded studies**<br>Enter a statement with the following details:<br>• Initials of the authors who received each award<br>• Grant numbers awarded to each author<br>• The full name of each funder<br>• URL of each funder website<br>• Did the sponsors or funders play any role in the study design, data collection and analysis, decision to publish, or preparation of the manuscript?<br><br>Did you receive funding for this work?"	UNITED STATES - US
**Competing Interests**	The authors have declared that no competing interests exist.

Use the instructions below to enter a competing interest statement for this submission. On behalf of all authors, disclose any competing interests that could be perceived to bias this work—acknowledging all financial support and any other relevant financial or non-financial competing interests.<br><br>This statement is required for submission and **will appear in the published article** if the submission is accepted. Please make sure it is accurate and that any funding sources listed in your Funding Information later in the submission form are also declared in your Financial Disclosure statement.<br><br>View published research articles from *PLOS ONE* for specific examples.<br><br>**NO authors have competing interests**<br><br>Enter: *The authors have declared that no competing interests exist.*<br><br>**Authors with competing interests**<br><br>Enter competing interest details beginning with this statement:<br><br>*I have read the journal's policy and the authors of this manuscript have the following competing interests: [insert competing interests here]*<br><br>* typeset	
**Ethics Statement**<br><br>Enter an ethics statement for this submission. This statement is required if the study involved:<br><br>• Human participants<br>• Human specimens or tissue<br>• Vertebrate animals or cephalopods<br>• Vertebrate embryos or tissues	This study received Institutional Review Board (IRB) approval from the São Paulo State Health Department (CAAE 12872419.6.0000.5469) and by the IRB at University of Nevada, Las Vegas (n. 1702327). The research committees of the participating municipalities and departments granted additional approvals. All participants provided verbal informed consent.

Powered by Editorial Manager® and ProduXion Manager® from Aries Systems Corporation

• Field research<br><br>Write "N/A" if the submission does not require an ethics statement.<br><br>General guidance is provided below. Consult the submission guidelines for detailed instructions. **Make sure that all information entered here is included in the Methods section of the manuscript.**	

Powered by Editorial Manager® and ProduXion Manager® from Aries Systems Corporation

**Format for specific study types**<br><br>**Human Subject Research (involving human participants and/or tissue)**<br>• Give the name of the institutional review board or ethics committee that approved the study<br>• Include the approval number and/or a statement indicating approval of this research<br>• Indicate the form of consent obtained (written/oral) or the reason that consent was not obtained (e.g. the data were analyzed anonymously)<br><br>**Animal Research (involving vertebrate animals, embryos or tissues)**<br>• Provide the name of the Institutional Animal Care and Use Committee (IACUC) or other relevant ethics board that reviewed the study protocol, and indicate whether they approved this research or granted a formal waiver of ethical approval<br>• Include an approval number if one was obtained<br>• If the study involved *non-human primates*, add *additional details* about animal welfare and steps taken to ameliorate suffering<br>• If anesthesia, euthanasia, or any kind of animal sacrifice is part of the study, include briefly which substances and/or methods were applied<br><br>**Field Research**<br><br>Include the following details if this study involves the collection of plant, animal, or other materials from a natural setting:<br>• Field permit number<br>• Name of the institution or relevant body that granted permission	
**Data Availability**<br><br>Authors are required to make all data underlying the findings described fully available, without restriction, and from the time of publication. PLOS allows rare exceptions to address legal and ethical concerns. See the PLOS Data Policy and FAQ for detailed information.	No - some restrictions will apply

A Data Availability Statement describing where the data can be found is required at submission. Your answers to this question constitute the Data Availability Statement and **will be published in the article**, if accepted.<br><br>**Important:** Stating 'data available on request from the author' is not sufficient. If your data are only available upon request, select 'No' for the first question and explain your exceptional situation in the text box.<br><br>Do the authors confirm that all data underlying the findings described in their manuscript are fully available without restriction?	
**Describe where the data may be found in full sentences. If you are copying our sample text, replace any instances of XXX with the appropriate details.**<br><br>• If the data are **held or will be held in a public repository**, include URLs, accession numbers or DOIs. If this information will only be available after acceptance, indicate this by ticking the box below. For example: *All XXX files are available from the XXX database (accession number(s) XXX, XXX.*).<br>• If the data are all contained **within the manuscript and/or Supporting Information files**, enter the following: *All relevant data are within the manuscript and its Supporting Information files.*<br>• If neither of these applies but you are able to provide **details of access elsewhere**, with or without limitations, please do so. For example:<br><br>*Data cannot be shared publicly because of [XXX]. Data are available from the XXX Institutional Data Access / Ethics Committee (contact via XXX) for researchers who meet the criteria for access to confidential data.*<br><br>*The data underlying the results presented in the study are available from (include the name of the third party*	A de-identified data set is not possible to provide due to ethical and legal considerations. These sharing restrictions are imposed by the UNLV Institutional Board Review (IRB). The authors declare that a de-identified data set from this study are available upon request directly to Dr. Buccini, assistant professor at UNLV (gabriela.buccini@unlv.edu) and/or to the UNLV IRB (irb@unlv.edu).

Powered by Editorial Manager® and ProduXion Manager® from Aries Systems Corporation

and contact information or URL).<br>• This text is appropriate if the data are owned by a third party and authors do not have permission to share the data.<br><br>* typeset	
Additional data availability information:	

Manuscript

Click here to access/download;Manuscript;Main Document_submited_references-IRB CORRECT.docx

Addressing Food Insecurity in Early Childhood Programs through a Health Equity Lens: A Qualitative Case Study of Brazil's *Criança Feliz* Program.

Gabriela Buccini[1&], Poliana de Araújo Palmeira[2*], Ana Poblacion[3π], Muriel Bauermann Gubert[1,4*]

[1]Department of Social and Behavioral Health, University of Nevada, Las Vegas, NV, USA.

[2]Centro de Educação e Saúde, Universidade Federal de Campina Grande, Cuité, PB, Brasil.

[3]Boston Medical Center, Boston, MA, USA.

[4]Departamento de Nutrição, Universidade de Brasília, Brasília, DF, Brasil.

Correspondent Author: gabriela.buccini@unlv.edu.

[&]Conceptualization, Funding acquisition, Methodology, Supervision, Writing – review & editing

* Data analysis, Writing – review & editing

[π]Writing – review & editing

Abstract

Background: Food insecurity (FI) is a public health crisis that negatively impacts early childhood health and development. Recommendations for establishing equitable actions to address FI in early childhood programming have not been developed globally. The Brazilian *Criança Feliz* Program (PCF), one of the largest early childhood home visiting programs worldwide, has faced implementation challenges while addressing FI. We aimed to (1) analyze programmatic barriers to addressing FI and (2) develop equity-focused recommendations to improve program implementation to mitigate FI.

Methods: Qualitative case study analyses of in-depth interviews with PCF implementation teams and families in five Brazilian municipalities. Participants shared their experience with PCF implementation quality, including questions related to FI. A three-stage rapid qualitative approach was used: (1) inductive thematic analysis identified central codes related to FI, (2) deductive approach to organize central codes within themes related to internal and external contexts of PCF operations, and (3) integration of findings into a set of equity-focused recommendations based on the four dimensions of the Getting to Equity (GTE) framework.

Results: 240 interviews were conducted. Internal programmatic barriers included lack of protocols for screening, referring, and following up with families struggling to access food as well as challenges to engage them in early learning activities. External programmatic barriers included family-level challenges (e.g. unrealistic support expected from PCF) and system-level challenges (e.g. bureaucracy in accessing safety nets). Ten equity-focused recommendations across GTE framework focused on improving program curriculum and protocols to mitigate FI and increasing individual and community capacity.

Conclusions: We documented barriers at the family, program, and system levels to address FI in the Brazilian PCF home visiting program. Barriers informed the generation of equity-focused programming recommendations to improve practices to address FI, not only for the PCF, but also for the global community implementing home visiting programs.

Introduction

Food insecurity (FI) is characterized by the lack of regular access to enough safe and nutritious food for normal growth and development and an active and healthy life, due to lack of resources to obtain food[1, 2]. The presence, severity, and persistence of FI have been associated with poor early childhood development, including delays in the academic, cognitive, behavioral, and socioemotional domains[3, 4]. The association between FI and early childhood development can occur through different mechanisms. A well-documented mechanism is through inadequate nutrition whether by the lack of adequate quantity or low-quality of food to ensure the nutritional needs for optimal development are met[5]. Another mechanism is the psychosocial stresse[5] such as caregiver's anxiety or depression[6]. Stress reduces opportunities for early stimulation (e.g. exposure to educational books, games, and toys)[7] as well as the ability of caregivers to provide responsive care, which in turn undermine optimal early childhood development[8]. Therefore, FI is a sensitive measure of inequities in early childhood development contributing to the perpetuation of the cycle of poverty[4].

In Brazil, the largest country in Latin America and the Caribbean with more than 215 million inhabitants, 27.6% of households were food insecure in 2023[9]. Households with children were disproportionately impacted by FI with 66.1% of households experiencing FI and 18.1% severe FI, in which children are hungry[9,10]. Thus, addressing FI in households with children has been a complex problem for decision-makers in Brazil, as no children should go hungry. FI

Previous complex systems dynamics analysis identified at least two types of leverage points to address FI in households with children[11] : (1) national/local policy interventions that affect the context in which the household operates and (2) interventions that directly affect household and caregiver options for action upon FI[11,12]. Specifically, a leverage intervention to reduce FI

and promote early childhood development is participation in early childhood home visiting programs[11].

The *Criança Feliz* Program (PCF) is one of the largest early childhood home visiting programs of the world surpassing 57 million home visits across 3,028 Brazilian municipalities (out of 5, 570 municipalities)[13] (see S1 Table for program description). Accordingly, the five components of the Nurturing Care Framework (NCF) (i.e., health, nutrition, early learning, security and safety, and responsive caregiving) have been a useful roadmap to design a multisectoral approach to address FI in the context of early childhood development[14]. Yet, several challenges in operationalizing the nurturing care multisectoral approach under the PCF have been documented at national, state, and municipal levels[12,15]. The PCF has fallen short in promoting early childhood development due to the lack of implementation quality goals in the initial seven years of scaling up (from 2016 to 2022)[12, 15-17]. One aspect hindering implementation quality was poor multisectoral approach to addressing social determinants of health such as FI[12], [18]. In 2023, after PCF reached its full implementation, the program was incorporated into the Brazilian Unified Social Protection System[19]. So, opportunities arise from investing efforts to optimize the nurturing care multisectoral approach to address FI among households with young children enrolled in PCF, likely yielding major social returns. For that, evidence-informed recommendations for establishing equitable actions to address FI in early childhood programming need to be developed for Brazil and lessons shared globally. The Getting to Equity (GTE) is a public health nutrition framework used to design recommendations to increase the impact of food security programs[20-22]. However, to our knowledge, no study has applied the GTE framework to develop equity-focused recommendations for addressing FI in early childhood programs. Thus, we aimed to (1) analyze programmatic barriers and facilitators

to addressing FI in families with young children enrolled in PCF and (2) integrate these findings to develop equity-focused recommendations to improve program implementation.

Methods

This study received Institutional Review Board (IRB) approval from the São Paulo State Health Department (CAAE 12872419.6.0000.5469) and by the IRB at University of Nevada, Las Vegas (n. 1702327). The research committees of the participating municipalities and departments granted additional approvals. All participants provided verbal informed consent. The Consolidated Criteria for Reporting Qualitative Studies (COREQ)[23,24] was followed to report results of this manuscript (S2 Table).

Study context

This exploratory qualitative case study[25] used in-depth interviews conducted with implementation teams and families participating in PCF for at least 6 months in five Brazilian municipalities. Due to the exponential increase of FI during the COVID-19 pandemic, interview guides were intentionally developed to include questions about FI and nurturing care multisectoral approach. Responses to these questions informed data analysis presented in this manuscript.

Selection of Participants

Purposive sampling was used to select information-rich municipality cases for the most effective use of limited evaluation resources[26]. Inclusion criteria to select five Brazilian

municipalities were (1) population size, (2) implementation length, (3) implementation model, and (4) geographical region. Four large urban municipalities of Campo Grande-MS, Brasília-DF, Fortaleza-CE, and São Paulo-SP were selected predominantly due to the challenges of implementing PCF in large urban centers[15], and one small predominantly rural municipality (Cuité-PB) was selected for analytical comparison. These municipalities are situated in the North/Northeast (n=2), Central-west (n=2), and South/Southeast (n=1), covering all Brazilian geographical regions.

In each municipality, participants were teams implementing PCF (i.e., home visitors, supervisors, municipal managers, and managers from different sectors interacting with PCF) and families participating in the PCF, both for at least 6 months. The sample size was designed to reach thematic saturation per municipality; therefore, in each municipality, families with different characteristics (e.g. pregnant, caregivers of children under 36 months, child beneficiaries of Cash Transfer Programs) were intentionally interviewed. In-depth interviews were conducted virtually via computer or phone with a sample of (a) municipal PCF teams, including municipal coordinators, supervisors, and home visitors (N = 132); (b) municipal managers from other sectors working on the implementation of the PCF, such as health, education, and social assistance (N = 17); and (c) families participating in the PCF (N = 95) (participant characteristics detailed in S3 and S4 Tables). No participants refused to participate. The data were collected from 01-06-2021 to 15-05-2022. .Full description of the selection of municipalities and participants is described elsewhere[12].

Research Team's Positionality

Interviews were conducted in Portuguese by trained native Portuguese speaker women researchers (GB, MBG, PPS). Data analysis of the FI component of interviews was conducted by faculty members and co-authors trained in public health nutrition and with experience in research on FI and early childhood in Brazil and the U.S. They all had prior experience conducting qualitative interviews and had no prior relationship with the participants. Participants were told the goals of the study prior to the interview.

Data management

Interviews were conducted throughout the months of June 2021 and May 2022 when physical distancing measures against COVID-19 pandemic were loosened. Debriefing meetings were held at the end of each day of interviews to expand field notes and conduct preliminary data coding and analysis. Through this approach, when thematic saturation was achieved in each municipality, the sample size was deemed sufficient for that municipality[27]. Interviews lasted 40–70 min each, were audio recorded with permission, and transcribed verbatim by a professional Portuguese-speaking service. Original audio recordings were compared to transcripts to ensure accuracy.

Interview guides

Interview guides were developed to capture PCF implementation quality barriers and facilitators. In the interview guide for PCF team and managers, questions followed the RE-AIM Framework dimensions, i.e., Reach, Effectiveness, Adoption, Implementation, Maintenance[28,29]. Interview guides were customized to address the specific needs of different program audiences, resulting in two distinct versions pre-tested with a member of the PCF

municipal team and a family member. Pertinent to this study, specific questions about programming approaches to address FI were taken from the implementation dimension. In the interview guide for families, questions followed the Nurturing Care Framework dimensions[30]. Pertinent to this study, specific questions about FI and participation in social protection programs in addition to the PCF were taken from the adequate nutrition dimension (S5 Table). Both interview guides had a section on sociodemographic characteristics of participants, which included the two-item screening tool (TRIA) to identify households at risk for food insecurity[31]. When a participant caregiver responded affirmatively to both items of the instrument, their household was considered at risk for food insecurity[31].

Data Analysis

Rapid qualitative methods, defined as research designed to be responsive and adaptable to changes in context while the study was ongoing[32], were used in three stages to analyze data (Figure 1)[33]. This methodology has been widely used in the field of implementation science to expedite knowledge translation and inform policy decisions[32,34].

In the first stage of thematic analysis, two researchers listened to interviews' audios and recorded information in a predefined matrix to capture barriers and facilitators to addressing FI throughout PCF implementation. A third researcher compared and validated the information recorded. Questions were discussed and confirmed throughout revisiting original audios. Then, an open-coding process[33] as used to inductively label barriers and facilitators to addressing FI. During this stage, frequency, complementarity, expansion, and divergence of coded information were considered to identify central codes of barriers and facilitators for implementation quality[33,34]. Several central codes related to FI emerged as barriers to the implementation

quality of the PCF[12] (Figure 1). The preliminary findings were presented to each municipality for feedback, fostering reflection within the research team and guiding the integration of data included in this manuscript.

Fig 1. Stages of the thematic data analysis

In the second stage of thematic analysis, in-depth analyses were conducted to understand how FI impacts the internal and external contexts of PCF operations. Thus, the central codes related to FI identified in phase 1 of data analysis were deductively grouped into two major themes by similarity, complementarity, and meaning as follows: (1) internal context in which PCF operates (i.e., program implementation and curriculum) and (2) external context in which PCF operates (i.e., families and systems). Quotes from interviews that best represented themes were selected to illustrate the results and were translated from Portuguese (Figure 1).

In the third stage of thematic analysis, GTE framework was used to reach consensus on integration of findings into a cohesive and pragmatic set of equity-focused recommendations to navigate FI in the context of early childhood programming (Figure 1). The premise for using GTE is that disparities in early childhood development cannot be overcome without addressing underlying inequities such as FI[3,4]. The GTE framework has been designed to provide practitioners and researchers with strategies to increase equity in policies, systems, and environmental change interventions that aim to decrease obesity[20,21], and FI[22,35]. Due to the lack of recommendations to address FI in early childhood programs, the GTE definitions were adapted for the four dimensions to support the development of equity-focused recommendations: (1) reduce deterrents (i.e., PCF strategies to screen and manage FI), (2)

increase healthy food (i.e., PCF strategies to improve access to healthy food), (3) improve social and economic resources (i.e., PCF strategies to alleviate FI in partnership with other programs), and (4) build community capacity (i.e., strategic choice of community partnerships to alleviate FI) (Figure 2).

Figure 2. Adapted definitions for each dimension of the Getting to Equity Framework to support the development of equity-focused recommendations for early childhood programming

Results

A total of 147 in-depth interviews with PCF teams, including municipal coordinators (n=38), supervisors (n=30), home visitors (n=69), and municipal managers from sectors such as social protection, health, and education working directly with PCF teams (n=10) were conducted across five Brazilian municipalities. The majority of professionals self-identified as females (89.1%), black or brown skin color (61.9%), older than 42 years (40.1%), having college education (49.0%), and 1 to 4 years of experience implementing programs (38.1%). A total of 93 in-depth interviews with families were conducted across five municipalities. The prevalence of FI risk in households with children interviewed for this study ranged from 31.6% in Fortaleza to 73.3% in Campo Grande. Among caregivers interviewed, the majority self-identified as having black or brown skin color (83.0%), not employed at time of interview (70.2%), and being beneficiaries of the Brazilian conditional cash transfer program Bolsa Familia (69.9%) (S3-S4 Tables).

Table 1 summarizes subthemes identified at the program, family, and system levels documenting the internal and external contexts in which PCF operates.

Table 1 Themes and subthemes associated with food insecurity in households with children enrolled in the Brazilian Criança Feliz Program.

Themes	Level	Subthemes
The internal context in which PCF operates	Program	• High frequency and levels of food insecurity among PCF families
		• Challenges to engage food-insecure families in early learning activities
		• Lack of official guidance on how to navigate food insecurity
		• Lack of formal resolution for severe levels of food insecurity
The external context in which PCF operates	Family	• Expectations of stipends or food baskets tied to PCF participation
		• Prioritization of PCF families to receive a one-time food stipend
		• Participation in the national conditional cash transfer program
	System	• Lack of social assistance sector preparedness
		• Daycare can be a protective factor against severe food insecurity
		• Informal network among PCF home visitors and local/community resources

The internal context in which PCF operates

All interviewees reported that the COVID-19 pandemic exacerbated existing economic and social vulnerabilities leading to **high prevalence of FI risk among households participating in PCF**. Several families participating in PCF described challenges in accessing food due to limited income and high food prices. Specifically, caregivers shared that the uncertainty of having enough food was a major household stressor, which strained the relationships in the household, as described by a mother participating in the PCF:

> Here our financial conditions to buy everything to eat is difficult, it's a little tight, isn't it? (...) To be honest, I don't remember (...) when I last went to the store to buy lettuce or squash, or okra, a maxixe, I don't remember when I went (…) It's

> pretty bad [dealing with FI]. Sometimes I get stressed. I take it out on my husband, but it's not his fault, is it? Thank God he works. But... things these days are so expensive. Everything is difficult (caregiver, Campo Grande).

Home visitors found it **challenging to engage caregivers struggling with food in early learning activities** such as play, games, and fun activities. They questioned whether teaching these activities should be a priority when people may be experiencing hunger. From their perspective, these activities designed to primarily strengthen the bond between dyads caregiver-child to promote early learning are neither feasible nor appropriate in the context of many competing social needs. In this context, from the perspective of home visitors and families, the effectiveness of learning activities available from PCF was undermined by the FI experience, as emphasized by a home visitor:

> Many [of the caregivers] do not see this positive side [in a child's early learning provided by the PCF]. It's very difficult to make a family that is often hungry understand the objective [of the PCF], what's gained in learning, [what is the point] of playing with the child (home visitor, São Paulo).

When asked about protocols to assist households at risk for FI to access food, home visitors and supervisors reported the **absence of a government official guidance on how to navigate FI** within PCF. The most immediate response from PCF teams to address FI was finding ways of accessing food baskets to donate to families in need. However, PCF teams reported that the supply of food baskets was insufficient to meet the demand from families in need, as reported by a home visitor:

> The difficulty we have at the moment is to meet people's demands because hunger is constant and food is a daily necessity. (…) We have a low supply [of basic food baskets] and a huge demand. There's never enough money (…). And that's what makes everyone anxious (…), but often our hands are tied, because we can't help all the families. (…) It's very complicated. (home visitor, Cuité).

Despite the lack of specific official guidance to manage screening and referral of families reporting having difficulties accessing food, home visitors used available resources to mitigate the issue. For example, they mentioned getting food baskets to households, which involves connecting families with official social protection services. The process of connecting with social protection services was described as bureaucratic, hierarchical, exhausting, and many times did not result in solutions for families, as highlighted by a home visitor:

> The person needs a food basket (…) You have to make a request, and you go to the supervisor, and the supervisor goes to I don't know where and nothing happens. When you go to see the person, they haven't even received anything (home visitor, Campo Grande).

The lack of resources and guidance generated a lot of frustration among home visitors when working with food-insecure households. Indeed, some home visitors shared that **when no formal resolution for cases** was found through the official social protection services, they used their personal or collective financial resources to purchase food baskets for families, as stated by a home-visitor:

> When you try to get a basket and you can’t find it. Then, you have to do it yourself. But sometimes you also do not have the money to buy it, because nowadays everything is very expensive. Then I started asking [colleagues to chip in with money] to buy a food basket (…) (home visitor, Brasilia)

Although reporting informal resolutions, home visitors understood these were not long-term solutions for families. However, most of them shared that in critical situations like hunger, they could not move on with their day without providing any type of support to the families.

The external context in which PCF operates

Family level. Families assisted by the PCF reported the COVID-19 pandemic as a major event exacerbating economic strains (e.g. lack of money for food and other competing basic needs) and social vulnerabilities (e.g. unemployment, domestic violence, household stress). Coping strategies were also reported to manage strains and vulnerabilities. Specifically, families struggling with food were prone to ask for help from other people to get food, skip meals or not eat meals to save food for their children's next meal. When families were asked whether PCF could help them navigate FI, caregivers expressed the **expectation that PCF would provide stipends or food baskets tied to program participation**, as illustrated by the following quote:

> How difficult is that? Because we don’t have any benefits [participating in PCF], and we eat what we have, [with the] help of other people, so we have to save as much as possible, especially for our children, we stop eating to give to our children (…) [The PCF] could help me get something. Especially with food stamps, right? With gas, everything helps… (mother, Fortaleza).

It is important to acknowledge that the families' expectations were unrealistic since there is no financial assistance tied to PCF participation. However, supervisors and home visitors from one municipality shared a unique experience that proved two opposite points. On the positive side, policies to mitigate FI can be rapidly enacted. On the negative side, rapidly enacted policies can pose a threat to governance. During the pandemic, a specific municipal government decided to **prioritize families participating in PCF to receive a one-time food stipend** (R$250 [~US$50]). PCF teams were responsible for confirming families that were actively participating in program activities and the social assistance secretary was responsible for registering eligible families in the food stipend program. However, this one-time effort to mitigate FI had limited coverage and left many families in need without access, which created confusion among families as to why some received the stipend and others did not, as highlighted in the quote from a home visitor:

> As the PCF team, we confirmed the list of people active in the program, including information on those who have already received the food stipend program, [those who] are no longer receiving it, and [those who] will receive again (…) The process that takes the longest to put these people in the food stipend program is the interview with the [social assistance] worker. Thus, the only thing we [as a PCF home visitor] can do for those asking about the food stipend program is to request [the families] to call again [to schedule an interview with the social assistance worker] (home visitor, Brasilia).

In a way or another, PCF families often found themselves without adequate support when experiencing FI. **Increasing family income through participation in the national conditional cash transfer program** (i.e., Bolsa Familia Program) emerged as a facilitator to reduce economic vulnerabilities, which in turn would directly affect FI. Despite that, several home visitors expressed concerns about the COVID-19 pandemic-era government that changed some of the eligibility criteria of Bolsa Familia, reducing the number of eligible families. From the perspective of those interviewed, this was worrying because it could increase FI among children. Specifically, families stated that the monthly cash transfer was used to buy food for their children, which in turn shielded them from FI, as reported by a caregiver:

> My husband is unemployed, we are surviving, getting hourly day work here and there; then he buys a little something, however, our child's food is specifically [bought with money] from Bolsa Familia. I just buy her food, and the rest of us make do (caregiver, Cuité).

System level. PCF managers recognized challenges in promoting sustainable solutions to mitigate FI due to **bureaucratic processes in accessing government safety net (i.e., social protection services) as well as limited non-government safety net (i.e., community resources)**. At the time of data collection, when families shared with home visitors, they are experiencing hunger, particularly not having food for the day; then, home visitors would notify supervisors, who in turn would notify PCF managers. Managers were able to refer these families to municipal social assistance centers. These municipal social assistance centers are responsible for assessing the needs of each family and enrolling them in as many social programs as they are eligible for such as Bolsa Familia, as seen in a home visitor's statement:

> "So, we bring to municipal social assistance centers [the needs of each FI family]. Then municipal social assistance centers within the social assistance sector make the move to call families and schedule a time for them to attend an in-person meeting at the municipal social assistance center. When families attend this meeting, they [families] receive what they need for example a food basket, rental assistance, and sometimes, even financial assistance with medication, when the family is very vulnerable, because many are. So, everything comes through the municipal social assistance centers" (home visitor, Cuité).

According to the families and home visitors interviewed, there was a huge waitlist with waiting times varying from one to seven months or more to schedule an initial interview, to then be able to access the services offered by the municipal social assistance centers. These waiting times were reported to be increased due to the high demand during COVID-19. The slow and bureaucratic process to get enrolled into the municipal social assistance centers resulted in families not having prompt access to social assistance programs to help them with economic vulnerabilities. The bureaucratic process was described by the interviewees as a lack of system preparedness during the pandemic. All interviewees agreed that the municipal social assistance sector had limited resolutions to address timely the complexity of FI, as one manager noted:

> "It's noteworthy. The problem of food insecurity exceeds what the social assistance sector can offer at all levels (…) both the municipal social assistance centers and our [social assistance] sector are not prepared and cannot address this complex issue of food insecurity" (PCF manager, São Paulo).

In this context, other community resources such as **daycare emerged as protective factors against hunger** among young children participating in the PCF. Many families stated that their children depended on the food provided by daycare centers. Thus, the shutdown of daycares during the COVID-19 pandemic meant that their children consumed less food. Therefore, the return of daycare operations was considered by all interviewees a positive measure to mitigate FI among young children participating in the PCF, as highlighted by a caregiver:

> "(…) now that school [daycare] is back on, children aren't so bad off. Because they have food at school. But it's lunch and dinner. Sometimes there's no breakfast, no snack. Understand?" (caregiver, Brasilia)

In search for additional resources to support food-insecure households, home visitors reported engaging informally with community organizations, including non-profit neighborhood organizations, churches or religious places, and local grocery shop owners. Home visitors shared that **building this informal/non-governmental network** was strategic particularly during the COVID-19 pandemic as they used it to obtain donations of food baskets to families in urgent need of food, as highlighted in a home visitor quote:

> "(…) We do it like this: in this case, I seek out [to build a relationship with a] neighborhood association (…) When I notice that a family is in a very vulnerable situation, then I ask [to the neighborhood association] if they have a food basket to give [to that family in need], understand? (home visitor, Fortaleza)

Home visitors acknowledged that many times they were not able to obtain food baskets through these informal networks, which also depend on outside donations. On one hand, building this informal network was valuable for their work. On the other hand, the informality of these relationships made it hard to have it as a sustainable option for food-insecure families.

Data integration to inform equity-focused recommendations

Grounded in GTE, the recommendations related to the internal context included strategies to **reduce deterrents** to program implementation (e.g., staff training, development of protocols to screen, refer, and follow up with families at risk for FI, protocols for immediate access to food in case of hunger), and **increase access to healthy food** by tailoring program curriculum (e.g., address fear of discrimination, and share with families healthy food options) (Table 2).

Table 2. Equity-focused strategies (not exhaustive) for addressing food insecurity in early childhood home visiting programs

Getting to Equity (GTE) dimensions		Programming Recommendations for early childhood home visiting programs
Potential interventions in program implementation and curriculum	Reduce Deterrents (program implementation)	• Staff trained on the impacts of food insecurity on mental health, infant feeding, and early childhood development;
		• Create protocols to screen, refer, and follow up with families at risk for food insecurity, including why, how, and what;
		• Create protocols for immediate access to food in case of moderate and severe levels of food insecurity;
	Increase access to healthy food (program curriculum)	• Address fear of discrimination and educate/motivate families on healthy options;
		• Promote healthy behaviors by providing culturally tailored nutrition information;
Individual and community resources and capacity	Improve social and economic resources (families)	• Support families to seek ways of increasing household minimum wage through enrolment in social or food assistance programs;
		• Assist caregivers with child and family needs by sharing community resources and other programs;

	Build on community capacity (system)	• Create protocols to facilitate warm handoff to services aiming at reducing family’s social and economic vulnerabilities;
		• Document and leverage existing multi sectoral community assets to support food-insecure families such as daycares;
		• Build formal strategic partnerships to strengthen to facilitate access to healthy food for families in the program.

Recommendations related to the external context outline strategies to **improve social and economic resources for families** (e.g., support families' enrolment in social and/or food assistance programs, share resources and programs with families) and **build community capacity** to enhance the safety net system to promote food security (e.g., develop protocols and leverage community assets to address FI and other family vulnerabilities, and build partnerships) (Table 2).

Discussion

This study documented barriers and opportunities to equitably address FI among young children and their families enrolled in one of the largest early childhood programs worldwide. Within the internal context of PCF, high prevalence of FI associated with the absence of appropriate protocols to screen, refer, and follow up challenged the engagement of food-insecure households in early learning activities. Within the external context of the PCF, family-level barriers included expectations of receiving stipends or easy access to food baskets, and at the system level the timeliness, bureaucracy, and lack of preparedness of government and non-government organizations to support food-insecure households in face of a global pandemic.

These barriers were found to impact early learning and responsive care environments possibly due to food and nutrition restrictions[36], parental depression, stress, and anxiety[37], which in turn, may increase inequities in early childhood development and perpetuate the cycle of poverty[3-5]. The documentation of these barriers underscored the critical need for equity-focused recommendations to address FI within ECD home visiting programs. This study findings informed the development of a not exhaustive list of equity-focused recommendations grounded in the GTE that can enhance ECD program implementation as well as individual and community resources and capacity (see Table 2).

Within the internal context of the PCF, this study identified high prevalence of households with young children at risk for FI ranging from 31.6% to 73.3%. These numbers corroborate with global trends for maternal-child populations during the COVID-19 pandemic[38-40]. Understanding FI as a transient phenomenon with most households moving in and out of food security as family circumstances change[41] is important to propose recommendations that allow time for vulnerable families with children to recover from severe disruptions. This is particularly concerning because food insecurity adversely impacts child health and development[3,4,36]. Therefore, specifying recommendations to enhance the implementation capacity of ECD programs to support families in transitioning out of FI and sustaining food security status is crucial.

Protocols to regularly screen, refer, and follow up households at risk for FI can reduce deterrents to program implementation. Incorporating FI screening as a routine during early childhood home visits can be a low-burden strategy to establish continuity of care. Adoption of brief screening tools such as the validated two-item food insecurity screen (TRIA)[31] could be used to identify households at risk for food insecurity. In case of positive screens, ECD home

visiting teams would rely on multidisciplinary services personnel to classify the severity level of FI through a diagnosis scale such as the 14-item Brazilian Food Insecurity Scale (EBIA)[42] which is based on the Household Food Security Survey Module (HFSSM)[43]. The identification of FI severity level is critical to establish a targeted action plan and ensure equitable resource allocation. Therefore, the development of ECD home visiting program protocols would benefit from the inclusion of decision-making trees with (1) incorporation of a validated screening tool, (2) classification of FI severity level, (3) determination of a referral pathways for each severity level of FI, and (4) inclusion of messages/actions based on the governance of the ECD program.

Tailoring short-, medium-, and long-term messages/actions to the PCF curriculum is essential for improving the impact on ECD outcomes. While addressing FI, there is a specific opportunity to enhance the ECD curriculum by integrating evidence-informed messages on nutrition literacy, including food access and healthy eating. Evidence from programs implemented in middle- and low-income countries demonstrated that integrating nutrition messages into ECD programs is effective[44,45]. The integration of messages requires an understanding of how nutritional contexts and caregiver characteristics such as well-being, knowledge, and behavior may influence early child nutrition outcome[44]. Accordingly, as part of the implementation plan, ECD home visiting teams should be trained in the integrated curriculum and protocols to address FI. In addition, they should be educated about their role in supporting families navigating FI. Training and supportive supervision are important implementation strategies to reduce stress and retain the workforce of ECD programs[46]. Indeed, training ECD home visiting teams to recognize food deprivation is critical to reducing social disparities[47].

Within the external context of the PCF, family-level barriers included expectations of receiving stipends or facilitated access to food baskets tied to PCF participation. While these expectations were not met by the PCF in Brazil, our findings documented the efforts of PCF teams to enroll families in Bolsa Familia as one strategy to reduce the risk for FI. There is strong evidence that combining benefits such as cash transfers or food benefits (e.g. food basket, food stamps) to vulnerable families enrolled in early childhood and parenting programs is beneficial to addressing FI as well as to reducing the inequality gap[48]. Several institutional ways for combining early childhood program and cash transfers programs have been documented globally, including *integrated* (i.e., the parenting intervention is managed by Bolsa Familia, *convergent* (i.e., different agencies explicitly combine efforts to bring the separate cash transfer and parenting programs to the same populations), *alignment* (i.e., the cash transfer and the parenting programs do not explicitly coordinate with one another but deliver interventions to similar if not the same populations), and *piggybacking* (i.e., the cash transfer is delivered through a separate established platform)[48]. This study findings indicate that in Brazil there is an alignment between ECD and conditional cash transfer programs as both programs target similar populations. However, a convergent approach where both programs are integrated and mutually supportive could address risk for FI, additional social needs, and enhance the impact on ECD outcomes, as demonstrated in other middle-income countries with similar governance systems to Brazil[49].

This study findings documented system level barriers such as the timeliness and bureaucracy related to the scarce government and non-governmental social safety net to support food-insecure households with children. There is strong evidence that both government and non-government safety nets can play a key role in protecting against FI while families cope with the

effect of additional social needs. Government safety nets may include participation in government benefits such as food[50] assistance or cash transfer programs as discussed above. Additionally, results showed participation in school/daycare programs was one important mechanism in the government safety net to support food-insecure households with children. Evidence shows that universal and free school meals contribute to children's nutrition and households' food security[51]. Brazil's early education and care system provides universal free-of-cost daycare for about 50% of the population[52]. However, due to the COVID-19 pandemic, daycares shut down. Without the food support[53], several PCF families struggled to provide food for their children. As a result, PCF teams experienced pressure to fill the gap left by the government safety nets. Thus, this study documented strategies to build a non-governmental safety net to support FI households with children. Non-governmental partnerships with community-based non-profit organizations can be a successful strategy for immediate relief of hunger, and it has been a mechanism for civil society to engage in the fight to end hunger in Brazil since the 90's[54]; however, food nonprofits need more government support to be sustainable[55]. This will require national and local political efforts to establish and sustain continuity of care to navigate FI[56]. Recent cross-sectoral collaborations involving public and private institutions, such as the Pact Against Hunger are good examples of strategic and sustainable partnerships aiming to eradicate hunger in Brazil[57]. Formalizing strategic partnerships between ECD programs and multisectoral safety nets acknowledges that addressing children's food and nutrition needs is critical to achieving equity in ECD.

This study's limitation includes a small sample of Brazilian municipalities. Specifically, the selection of four state capitals and one small municipality left medium-sized municipalities unrepresented. Despite that, implementation challenges to address FI were similar in the included

municipalities, suggesting that findings may have captured program, families, and system barriers that are likely to be encountered across municipalities independent of population size. Additionally, the inclusion of interviews with PCF teams and families across targeted groups (i.e., pregnant women, children younger than three years) allowed for data saturation on challenges to address FI at the program, families, and system levels[27]. As a result, these findings and recommendations reflect themes that emerged from experiences of interviewees involved in implementing the PCF in urban and rural contexts across different Brazilian regions.

This study's strengths include the use of rapid analysis which demonstrated solid and replicable results compared to traditional in-depth qualitative coding analysis[32,58]. Thus, this approach is not only innovative but also provides timely knowledge translation into Public Health recommendations. The deductive application of the GTE framework to integrate findings into practical programming recommendations is also a strength. On the other hand, we acknowledge that the non-exhaustive list of recommendations outlined in this study represents and it is limited to the perspective of the co-authors, who are researchers working with evaluation of implementation programs related to FI and/or ECD in Brazil for several years. Therefore, the non-exhaustive list of recommendations is informed by this study's findings through the lens of their lived experiences and aligned with the most current literature on these topics. GTE has been extensively used to analyze dimensions of equity in obesity prevention[20] and access to healthy food[59] programs and in this study was instrumental in guiding specific and relevant programming recommendations to address FI within ECD programming to achieve equity in early childhood development.

In conclusion, this study documented barriers at the program, families, and system levels to address FI among households with young children enrolled in the Brazilian ECD home

visiting program in five Brazilian municipalities. Grounded in the GTE framework, this study integrated opportunities identified in actionable equity-focused programming recommendations that may enhance effectiveness of one of the largest ECD programs worldwide (see Table 2). During this period of reorienting PCF implementation, it will be crucial to consider the following key actions: (1) incorporate universal screening for risk of FI along with protocols to empower PCF teams to navigate FI, (2) develop messages to enhance the PCF curriculum on child food and nutritional needs and its impact on ECD, (3) enhance capacity of government safety net programs, including Bolsa Familia, food benefit programs (e.g. food basket, food stamps), and early education and care through a convergent integrated approach to address the FI, (4) establish a local non-governmental safety net by formalizing multisectoral partnerships to facilitate continuity of care for FI households and promote equity in ECD.

Acknowledgement

We would like to thank Dr. Sonia Isoyama Venancio, and research assistants Lidia Godoi and Laura Dal'Ava dos Santos for their assistance and support during this study.

References

1. Brasil. Presidência Da República. Lei No 11.346, de 15 de Setembro de 2006. Cria o Sistema Nacional de Segurança Alimentar e Nutricional (SISAN) Com Vistas Em Assegurar o Direito Humano à Alimentação Adequada e Dá Outras Providências. Diário Oficial Da União. Brasília; 2006.

2. Food and Agriculture Organization of the United Nations (2012) Coming to Terms with Terminology: Food Security, Nutrition Security, Food Security and Nutrition, Food and Nutrition Security. Rome, Italy: FAO.

3. Gallegos D, Eivers A, Sondergeld P, Pattinson C. Food Insecurity and Child Development: A State-of-the-Art Review. IJERPH. 2021;18(17):8990. doi:10.3390/ijerph18178990

4. De Oliveira KHD, De Almeida GM, Gubert MB, et al. Household food insecurity and early childhood development: Systematic review and meta-analysis. Maternal & Child Nutrition. 2020;16(3):e12967. doi:10.1111/mcn.12967

5. Perez-Escamilla R, Pinheiro De Toledo Vianna R. Food Insecurity and the Behavioral and Intellectual Development of Children: A Review of the Evidence. Journal of Applied Research on Children: Informing Policy for Children at Risk. 2012;3(1). doi:10.58464/2155-5834.1071

6. Pedroso J, Herkrath FJ, Buccini G, Venancio SI, Pérez-Escamilla R, Gubert MB. Maternal Mental Health Mediates the Relationship Between Household Food Insecurity and Early Child Development in Brazil. Food Nutr Bull. 2021;42(1):77-90. doi:10.1177/0379572120987815

7. Venancio SI, Teixeira JA, De Bortoli MC, Bernal RTI. Factors associated with early childhood development in municipalities of Ceará, Brazil: a hierarchical model of contexts, environments, and nurturing care domains in a cross-sectional study. The Lancet Regional Health - Americas. 2022;5:100139. doi:10.1016/j.lana.2021.100139

8. Johnson AD, Markowitz AJ. Food Insecurity and Family Well-Being Outcomes among Households with Young Children. The Journal of Pediatrics. 2018;196:275-282. doi:10.1016/j.jpeds.2018.01.026

9. Instituto Brasileiro de Geografia e Estatística (IBGE). Segurança Alimentar: 2023 [Internet]. Rio de Janeiro: Coordenação de Pesquisas Por Amostra de Domicílios; 2023 [Citado 24 Jul 2024]. Disponível Em: Https://Biblioteca.Ibge.Gov.Br/Index.Php/Biblioteca-Catalogo?

10. Rede Brasileira de Pesquisa Em Soberania e Segurança Alimentar – PENSSAN. II Inquérito Nacional Sobre Insegurança Alimentar No Contexto Da Pandemia Da COVID-19 No Brasil: II VIGISAN : Relatório Final. São Paulo, SP : Fundação Friedrich Ebert : Rede PENSSAN, 2022.

11. Buccini, G., Marcal, K., Gubert, M. B., Venancio, S. I., Perez-Escamilla, R., & Stave, K. (in Press). Causal Loop Diagram of Child Food Poverty During the First 1,000 Days in Brazil: A Nurturing Care Approach.

12. Buccini G, Gubert MB, De Araújo Palmeira P, et al. Scaling up a home-visiting program for child development in Brazil: a comparative case studies analysis. The Lancet Regional Health - Americas. 2024;29:100665. doi:10.1016/j.lana.2023.100665

13. Brasil. Ministério Do Desenvolvimento e Assistência Social, Família e Combate à Fome. Programa Criança Feliz Atinge Marca de Mais de 50 Milhões de Visitas Domiciliares. [Acessado Em 16 de Junho de 2023]. Disponível Em: Https://Www.Gov.Br/Mds/Pt-Br/Noticias-e-

Conteudos/Desenvolvimento-Social/Noticias-Desenvolvimento-Social/Programa-Crianca-Feliz-Atinge-Marca-de-Mais-de-50-Milhoes-de-Visitas-Domiciliares.

14. Buccini G, Saniatan KL, Poblacion A, et al. Nurturing care assets for food security: a community asset mapping approach. J Health Popul Nutr. 2024;43(1):78. doi:10.1186/s41043-024-00573-9

15. Buccini G, Venancio SI, Pérez-Escamilla R. Scaling up of Brazil's Criança Feliz early childhood development program: an implementation science analysis. Annals of the New York Academy of Sciences. 2021;1497(1):57-73. doi:10.1111/nyas.14589

16. Santos IS, Munhoz TN, Barcelos RS, et al. Avaliação do Programa Criança Feliz: um estudo randomizado em 30 municípios brasileiros. Ciênc saúde coletiva. 2022;27(12):4341-4363. doi:10.1590/1413-812320222712.13472022

17. Munhoz TN, Santos IS, Blumenberg C, et al. Fatores associados ao desenvolvimento infantil em crianças brasileiras: linha de base da avaliação do impacto do Programa Criança Feliz. Cad Saúde Pública. 2022;38(2):e00316920. doi:10.1590/0102-311x00316920

18. Dos Santos LMTD, Godoi L, De Andrade E Guimarães B, et al. A qualitative analysis of the nurturing care environment of families participating in Brazil's Criança Feliz early childhood program. Miranda AE, ed. PLoS ONE. 2023;18(7):e0288940. doi:10.1371/journal.pone.0288940

19. Brasil. Ministério Do Desenvolvimento e Assistência Social, Família e Combate à Fome (BR), Secretaria Nacional de Assistência Social. Resolução No 4, de 30 de Agosto de 2023. Consolida Os Atos Normativos Que Regulamentam o Programa Criança Feliz/Primeira Infância No Sistema Único de Assistência Social - SUAS. Diário Oficial Da União. 1o Set 2023;168(Seção 1):19.

20. Kumanyika SK. A Framework for Increasing Equity Impact in Obesity Prevention. Am J Public Health. 2019;109(10):1350-1357. doi:10.2105/AJPH.2019.305221

21. Drexel University, Kumanyika S. Getting to Equity in Obesity Prevention: A New Framework. NAM Perspectives. 2017;7(1). doi:10.31478/201701c

22. McLoughlin GM, McCarthy JA, McGuirt JT, Singleton CR, Dunn CG, Gadhoke P. Addressing Food Insecurity through a Health Equity Lens: a Case Study of Large Urban School Districts during the COVID-19 Pandemic. J Urban Health. 2020;97(6):759-775. doi:10.1007/s11524-020-00476-0

23. Souza VRDS, Marziale MHP, Silva GTR, Nascimento PL. Tradução e validação para a língua portuguesa e avaliação do guia COREQ. Acta Paulista de Enfermagem. 2021;34:eAPE02631. doi:10.37689/acta-ape/2021AO02631

24. Tong A, Sainsbury P, Craig J. Consolidated criteria for reporting qualitative research (COREQ): a 32-item checklist for interviews and focus groups. International Journal for Quality in Health Care. 2007;19(6):349-357. doi:10.1093/intqhc/mzm042

25. Yin RK. Case Study Research and Applications Design and Methods. SAGE Publications; 2017.

26. Palinkas LA, Horwitz SM, Green CA, Wisdom JP, Duan N, Hoagwood K. Purposeful Sampling for Qualitative Data Collection and Analysis in Mixed Method Implementation Research. Adm Policy Ment Health. 2015;42(5):533-544. doi:10.1007/s10488-013-0528-y

27. Morse JM. The Significance of Saturation. Qual Health Res. 1995;5:147–149.

28. Glasgow RE, Harden SM, Gaglio B, et al. RE-AIM Planning and Evaluation Framework: Adapting to New Science and Practice With a 20-Year Review. Front Public Health. 2019;7:64. doi:10.3389/fpubh.2019.00064

29. Holtrop JS, Estabrooks PA, Gaglio B, et al. Understanding and applying the RE-AIM framework: Clarifications and resources. J Clin Trans Sci. 2021;5(1):e126. doi:10.1017/cts.2021.789

30. Britto PR, Lye SJ, Proulx K, et al. Nurturing care: promoting early childhood development. The Lancet. 2017;389(10064):91-102. doi:10.1016/S0140-6736(16)31390-3

31. Poblacion A, Segall-Corrêa AM, Cook J, Taddei JADAC. Validity of a 2-item screening tool to identify families at risk for food insecurity in Brazil. Cad Saúde Pública. 2021;37(6):e00132320. doi:10.1590/0102-311x00132320

32. Gale RC, Wu J, Erhardt T, et al. Comparison of rapid vs in-depth qualitative analytic methods from a process evaluation of academic detailing in the Veterans Health Administration. Implementation Sci. 2019;14(1):11. doi:10.1186/s13012-019-0853-y

33. Bradley EH, Curry LA, Devers KJ. Qualitative Data Analysis for Health Services Research: Developing Taxonomy, Themes, and Theory. Health Services Research. 2007;42(4):1758-1772. doi:10.1111/j.1475-6773.2006.00684.x

34. Aarons GA, Fettes DL, Sommerfeld DH, Palinkas LA. Mixed Methods for Implementation Research: Application to Evidence-Based Practice Implementation and Staff Turnover in Community-Based Organizations Providing Child Welfare Services. Child Maltreat. 2012;17(1):67-79. doi:10.1177/1077559511426908

35. McLoughlin GM, Kumanyika S, Su Y, Brownson RC, Fisher JO, Emmons KM. Mending the gap: Measurement needs to address policy implementation through a health equity lens. Translational Behavioral Medicine. 2024;14(4):207-214. doi:10.1093/tbm/ibae004

36. Poblacion AP, Cook JT, Marín-León L, et al. Food Insecurity and the Negative Impact on Brazilian Children’s Health—Why Does Food Security Matter for Our Future Prosperity?

Brazilian National Survey (PNDS 2006/07). Food Nutr Bull. 2016;37(4):585-598. doi:10.1177/0379572116664167

37. Cain KS, Meyer SC, Cummer E, et al. Association of Food Insecurity with Mental Health Outcomes in Parents and Children. Academic Pediatrics. 2022;22(7):1105-1114. doi:10.1016/j.acap.2022.04.010

38. Hadi Dehghani M, Karri RR, Roy S, eds. COVID-19 and the Sustainable Development Goals. Elsevier; 2022.

39. Pérez-Escamilla R, Cunningham K, Moran VH. COVID-19 and maternal and child food and nutrition insecurity: a complex syndemic. Maternal & Child Nutrition. 2020;16(3):e13036. doi:10.1111/mcn.13036

40. Ng Y, Chang M, Robertson M, et al. Food Insecurity During the First Year of COVID-19: Employment and Sociodemographic Factors Among Participants in the CHASING COVID Cohort Study. Public Health Rep. 2023;138(4):671-680. doi:10.1177/00333549231170203

41. Grineski SE, Morales DX, Collins TW, Rubio R. Transitional Dynamics of Household Food Insecurity Impact Children’s Developmental Outcomes. J Dev Behav Pediatr. 2018;39(9):715-725. doi:10.1097/DBP.0000000000000598

42. Segall-Corrêa AM, Marin-León L, Melgar-Quiñonez H, Pérez-Escamilla R. Refinement of the Brazilian Household Food Insecurity Measurement Scale: Recommendation for a 14-item EBIA. Rev Nutr. 2014;27(2):241-251. doi:10.1590/1415-52732014000200010

43. Bickel, Gary, Mark Nord, Cristofer Price, William Hamilton, and John Cook: Guide to Measuring Household Food Security, Revised 2000. U.S. Department of Agriculture, Food and Nutrition Service, Alexandria VA. March, 2000.

44. Hurley KM, Yousafzai AK, Lopez-Boo F. Early Child Development and Nutrition: A Review of the Benefits and Challenges of Implementing Integrated Interventions. Advances in Nutrition. 2016;7(2):357-363. doi:10.3945/an.115.010363

45. Maalouf-Manasseh, Z; Oot, L; Sethuraman, K. 2015. Giving Children the Best Start in Life: Integrating Nutrition and Early Childhood Development within the First 1,000 Days. Washington, DC: FHI 360/FANTA.

46. Buccini G, Kofke L, Case H, Katague M, Pacheco MF, Pérez-Escamilla R. Pathways to scale up early childhood programs: A scoping review of Reach Up and Care for Child Development. Ziauddeen N, ed. PLOS Glob Public Health. 2023;3(8):e0001542. doi:10.1371/journal.pgph.0001542

47. Andermann A. Taking action on the social determinants of health in clinical practice: a framework for health professionals. CMAJ. 2016;188(17-18):E474-E483. doi:10.1503/cmaj.160177

48. Arriagada AM, Perry J, Rawlings L, Trias J, Zumaeta M. Promoting Early Childhood Development Through Combining Cash Transfer and Parenting Programs. World Bank Group. Available at: Https://Documents1.Worldbank.Org/Curated/En/489331538646764960/Pdf/Promoting-Early-Childhood-Development-through-Combining-Cash-Transfer-and-Parenting-Programs.Pdf. Accessed August 26, 2024.

49. Fernald LCH, Kagawa RMC, Knauer HA, Schnaas L, Guerra AG, Neufeld LM. Promoting child development through group-based parent support within a cash transfer program: Experimental effects on children's outcomes. Developmental Psychology. 2017;53(2):222-236. doi:10.1037/dev0000185

50. Parker SW, Todd PE. Conditional Cash Transfers: The Case of Progresa/Oportunidades. Journal of Economic Literature. 2017;55(3):866-915. doi:10.1257/jel.20151233

51. De Amorim ALB, Dalio Dos Santos R, Ribeiro Junior JRS, Canella DS, Bandoni DH. The contribution of school meals to food security among households with children and adolescents in Brazil. Nutrition. 2022;93:111502. doi:10.1016/j.nut.2021.111502

52. Raikes A, Alvarenga Lima JHN, Abuchaim B. Early Childhood Education in Brazil: Child Rights to ECE in Context of Great Disparities. Children. 2023;10(6):919. doi:10.3390/children10060919

53. Rodrigues EC, Mendonça RDD, Camargo PP, Menezes MCD, Carvalho NCD, Meireles AL. Home food insecurity during the suspension of classes in Brazilian public schools due to the COVID-19 pandemic. Nutrition. 2022;93:111448. doi:10.1016/j.nut.2021.111448

54. Pinto CRJ. A sociedade civil e a luta contra a fome no Brasil (1993-2003). Soc estado. 2005;20(1):195-228. doi:10.1590/S0102-69922005000100009

55. Berner M, Ozer T, Paynter S. A Portrait of Hunger, the Social Safety Net, and the Working Poor. Policy Studies Journal. 2008;36(3):403-420. doi:10.1111/j.1541-0072.2008.00274.x

56. Ribeiro-Silva RDC, Pereira M, Campello T, et al. Implicações da pandemia COVID-19 para a segurança alimentar e nutricional no Brasil. Ciênc saúde coletiva. 2020;25(9):3421-3430. doi:10.1590/1413-81232020259.22152020

57. Instituto Pacto Contra a Fome. Estatuto Social. [Acesso Em 16 de Junho de 2023]. Disponível Em Https://Pactocontrafome.Org/.

58. Nevedal AL, Reardon CM, Opra Widerquist MA, et al. Rapid versus traditional qualitative analysis using the Consolidated Framework for Implementation Research (CFIR). Implementation Sci. 2021;16(1):67. doi:10.1186/s13012-021-01111-5

59. Cardarelli K, DeWitt E, Gillespie R, Norman-Burgdolf H, Jones N, Mullins JT. “We’re, Like, the Most Unhealthy People in the Country”: Using an Equity Lens to Reduce Barriers to Healthy Food Access in Rural Appalachia. Prev Chronic Dis. 2020;17:200340. doi:10.5888/pcd17.200340

Supporting information

Supplementary Table S1. Criança Feliz Program national guidelines following the Template for Intervention Description (TIDieR)

Supplementary Table S2. Consolidated criteria for reporting qualitative research checklist (COREQ)

Supplementary Table S3

Socio-demographic characteristics of the professionals interviewed in the *Criança Feliz Program* (PCF), 2021-2022.

Supplementary Table S4

Socio-demographic characteristics of the families interviewed in the *Criança Feliz Program* (PCF), 2021-2022.

Suplementary Table S5.

Selected questions of the Interview Guide related to Food insecurity, vulnerability, and equity, according to the RE-AIM Framework dimensions (Reach, Effectiveness, Adoption, Implementation, Maintenance)

Figure 1

Click here to access/download;Figure;Fig1_File.tiff

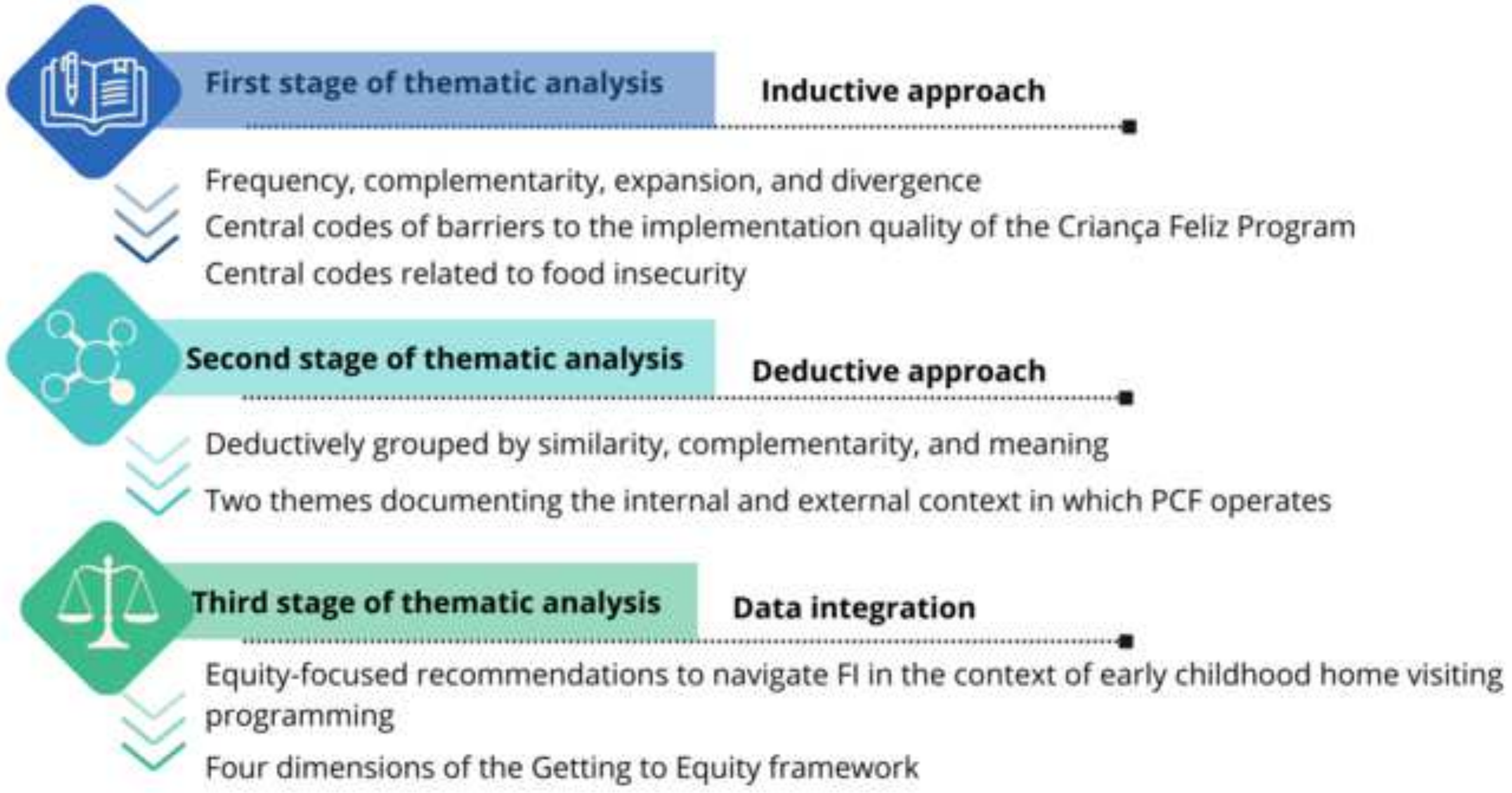

 Click here to access/download;Figure;S2_File.docx

Supplementary Table S2. Consolidated criteria for reporting qualitative research checklist (COREQ)

A checklist of items that should be included in reports of qualitative research. Below is reported the section where authors considered each of the items listed in this checklist. A note N/A was included when the information was not relevant.

Topic	Item No.	Guide Questions/Description	Reported on Section
**Domain 1: Research team and reflexivity**			
Personal characteristics			
Interviewer/facilitator	1	Which author/s conducted the interview or focus group?	Methods, Research Team Positionality
Credentials	2	What were the researcher's credentials? E.g. PhD, MD	Methods, Research Team Positionality
Occupation	3	What was their occupation at the time of the study?	Methods, Research Team Positionality
Gender	4	Was the researcher male or female?	Methods, Research Team Positionality
Experience and training	5	What experience or training did the researcher have?	Methods, Research Team Positionality
Relationship with participants			
Relationship established	6	Was a relationship established prior to study commencement?	Methods, Research Team Positionality
Participant knowledge of the interviewer	7	What did the participants know about the researcher? e.g. personal goals, reasons for doing the research	Methods, Research Team Positionality
Interviewer characteristics	8	What characteristics were reported about the interviewer/facilitator? e.g. Bias, assumptions, reasons and interests in the research topic	Methods, Research Team Positionality
**Domain 2: Study design**			
Theoretical framework			
Methodological orientation and Theory	9	What methodological orientation was stated to underpin the study? e.g. grounded theory, discourse analysis, ethnography, phenomenology, content analysis	Methods, Data Analysis
Participant selection			
Sampling	10	How were participants selected? e.g. purposive, convenience, consecutive, snowball	Methods, Selection of Participants

Method of approach	11	How were participants approached? e.g. face-to-face, telephone, mail, email	Methods, Selection of Participants
Sample size	12	How many participants were in the study?	Methods, Selection of Participants
Non-participation	13	How many people refused to participate or dropped out? Reasons?	Methods, Selection of Participants
Setting			
Setting of data collection	14	Where was the data collected? e.g. home, clinic, workplace	Methods, Selection of Participants
Presence of non participants	15	Was anyone else present besides the participants and researchers?	Methods, Selection of Participants
Description of sample	16	What are the important characteristics of the sample? e.g. demographic data, date	Supplementary Table S3 and S4
Data collection			
Interview guide	17	Were questions, prompts, guides provided by the authors? Was it pilot tested?	Methods, Interview Guides
Repeat interviews	18	Were repeat inter views carried out? If yes, how many?	N/A
Audio/visual recording	19	Did the research use audio or visual recording to collect the data?	Methods, Data management
Field notes	20	Were field notes made during and/or after the interview or focus group?	Methods, Data management

Duration	21	What was the duration of the inter views or focus group?	Methods, Data management
Data saturation	22	Was data saturation discussed?	Methods, Data management
Transcripts returned	23	Were transcripts returned to participants for comment and/or correction?	Methods, Data management
**Domain 3: analysis and findings**			
Data analysis			
Number of data coders	24	How many data coders coded the data?	Methods, Data Analysis
Description of the coding tree	25	Did authors provide a description of the coding tree?	Table 1

Derivation of themes	26	Were themes identified in advance or derived from the data?	Methods, Data Analysis
Software	27	What software, if applicable, was used to manage the data?	N/A
Participant checking	28	Did participants provide feedback on the findings?	Methods, Data Analysis
Reporting			
Quotations presented	29	Were participant quotations presented to illustrate the themes/findings? Was each quotation identified? e.g. participant number	Results
Data and findings consistent	30	Was there consistency between the data presented and the findings?	Results
Clarity of major themes	31	Were major themes clearly presented in the findings?	Results
Clarity of minor themes	32	Is there a description of diverse cases or discussion of minor themes?	Results

Developed from: Tong A, Sainsbury P, Craig J. Consolidated criteria for reporting qualitative research (COREQ): a 32-item checklist for interviews and focus groups. International Journal for Quality in Health Care. 2007. Volume 19, Number 6: pp. 349 – 357

Suplementary Table 1

Click here to access/download
Supporting Information
S1_File.docx

Suplementary Table 3

Click here to access/download
Supporting Information
S3_File.docx

Suplementary Table 4

Click here to access/download
Supporting Information
S4_File.docx

Suplementary Table 5

Click here to access/download
Supporting Information
S5_File.docx
