# Supplementary material for: Addressing food insecurity in early childhood programs through a health equity lens: A qualitative case study of Brazil’s Criança Feliz program
Source: PLoS One. 2025 Jul 28;20(7):e0329310. doi: 10.1371/journal.pone.0329310 (PMC12303329; doi:10.1371/journal.pone.0329310)
Supplement: Attachment — Submitted filename: FI_PlosOnr review 2.docx [file pone.0329310.s010.docx]

Dear Editor,

Thank you for the opportunity to improve the organization of our manuscript. Below are the response point-by-point to journal requirements and editor’s feedback. We hope our revised version meets your expectations.

=====================================================================

**Editor comments:**

PLOS ONE considers qualitative and mixed-methods studies for publication. We recommend that authors use the COREQ checklist, or other relevant checklists listed by the Equator Network, such as the SRQR, to ensure complete reporting (<http://journals.plos.org/plosone/s/submission-guidelines#loc-qualitative-research>).

Response: We used the COREQ checklist to report methods and results of this manuscript. This is indicated in lines 117-118 and the checklist is included as S2 Table.

In general, we would expect qualitative studies to include the following:

1) defined objectives or research questions;

Response: Defined objectives are indicated in lines 107-110 and 121-124.

2) description of the sampling strategy, including rationale for the recruitment method, participant inclusion/exclusion criteria and the number of participants recruited;

Response: The “Selection of Participants” sub-section in the Methods describe sampling strategy, including rationale for the recruitment method, participant inclusion/exclusion criteria and the number of participants recruited. We added sub-headings to help reader identify the two level of participants (i.e., municipalities, and implementation teams) included in the study. In addition, as specified in COREQ, we added the method of approach to participant selection. These changes are indicated in lines 132-163.

3) detailed reporting of the data collection procedures;

Response: We reorganized the headings in the “Methods” section to clarify the sub-sections of the data collection procedures. This is indicated in lines 165-203.

4) data analysis procedures described in sufficient detail to enable replication;

Response: We reorganized the headings in the “Methods” section to clarify the sub-sections of the data analysis procedures. This is indicated in lines 205-257.

5) a discussion of potential sources of bias;

Response: Limitations and potential sources of bias are presented between lines 600-616.

6) a discussion of limitations.

Response: Limitations and potential sources of bias are presented between lines 600-616.

=====================================================================

**Journal Requirements:**

Please review your reference list to ensure that it is complete and correct. If you have cited papers that have been retracted, please include the rationale for doing so in the manuscript text, or remove these references and replace them with relevant current references. Any changes to the reference list should be mentioned in the rebuttal letter that accompanies your revised manuscript. If you need to cite a retracted article, indicate the article’s retracted status in the References list and also include a citation and full reference for the retraction notice.

Response: No changes in the reference list were made at this time.
